# Harnessing genomic and bioinformatics for surveillance of pathogens in Africa: a scoping review of existing training and gaps in training

**DOI:** 10.1186/s12879-026-13671-2

**Published:** 2026-05-29

**Authors:** Paul Tunde Kingpriest, John Bok, Palpouguini Lompo, Evodie Ngelesi, Joseph Mwaka, Sainabou Laye Ndure, Nyambura Moremi, Vito Baraka, Tim Van Den Bossche, Sakina Bombaywala, Bart Mesuere, Lennart Martens, Trudie Lang

**Affiliations:** 1 The Global Health Network, Centre for Tropical Medicine and Global Health, University of Oxford, Oxford, UK; 2 Institut de Recherche en Sciences de la Santé – Clinical Research Unit of Nanoro (IRSS-CRUN), Nanoro, Burkina Faso; 3 Department of Tropical Medicine, University of Kinshasa, Kinshasa, Democratic Republic of the Congo; 4 National Public Health Laboratory, Dar es Salaam, Tanzania; 5 The National Institute for Medical Research, Tanga, Tanzania; 6 VIB – UGent Center for Medical Biotechnology, VIB, Ghent, 9052 Belgium; 7 Department of Biomolecular Medicine, Faculty of Medicine and Health Sciences, Ghent University, Ghent, 9000 Belgium; 8 Department of Mathematics, Statistics and Computer Science, Faculty of Sciences, Ghent University, Ghent, 9000 Belgium

**Keywords:** Genomics training, Bioinformatics, Pathogen surveillance, Capacity building, Africa

## Abstract

**Background:**

Pathogen surveillance is vital for managing infectious diseases in Africa, where high disease burdens necessitate advanced tools like genomics and bioinformatics. While these technologies enable early detection, resistance monitoring, and targeted interventions, yet their adoption is hindered by limited training and educational resources. This scoping review maps existing training programmes in genomics and bioinformatics for pathogen surveillance in Africa, identifying gaps and opportunities to enhance capacity-building.

**Methods:**

Following PRISMA-ScR guidelines, we systematically searched databases (PubMed, Embase, Scopus, etc.) and grey literature for articles which described the design, implementation, delivery, or evaluation of a specific genomics/bioinformatics training programme or discussed training needs or status published between November 2014 and December 2025. Eligible articles focused on genomics or bioinformatics training for pathogen surveillance in Africa. Data were extracted on programme characteristics, outcomes, challenges, and recommendations, with findings synthesised narratively and thematically.

**Results:**

Of 2491 identified articles, 40 were included, spanning 2014–2025. These comprised case studies (45%), opinion pieces (27.5%), reviews (15%), cross-sectional studies (10%), and a methodological study (2.8%). Twenty-three articles detailed specific training programmes, predominantly short-term (e.g., 2–14 days), with 60.9% of the training programmes covering both genomics and bioinformatics. Training programmes were mostly carried out in African countries (91.3%), often funded by external agencies like NIH (26.1%) and Wellcome Trust (13%). Key outcomes included enhanced technical skills (21.7%) and career development (17.4%). Challenges included inadequate infrastructure, skill gaps, and theoretical-heavy curricula, while opportunities encompassed virtual delivery, collaborative networks, and open-access resources. Seventeen studies were included as they examined training status and deficiencies in Africa, highlighting limited expertise and brain drain, and recommending investment in infrastructure, expansion of curricula, and development of local trainers.

**Conclusion:**

Genomics and bioinformatics training in Africa is growing but remains fragmented, donor-dependent, and resource-constrained. Sustainable capacity-building requires institutionalising training within academic systems, increasing government investment, and leveraging open-access and regional networks. These findings provide a foundation for policymakers and educators to develop targeted, inclusive programmes, strengthening Africa’s pathogen surveillance capabilities.

**Clinical trial number:**

N/A.

**Supplementary Information:**

The online version contains supplementary material available at 10.1186/s12879-026-13671-2.

## Background

Pathogen surveillance is a critical component of public health, enabling early detection, monitoring, and response to infectious diseases. Genomics and bioinformatics have revolutionised this field by providing high-resolution insights into pathogen evolution, transmission, and resistance patterns [1, 2]. Through whole genome sequencing (WGS) and computational analysis, researchers can track the emergence of novel pathogens, understand their genetic diversity, and design effective interventions [3, 4]. The application of these technologies is particularly crucial in regions with high infectious disease burdens, such as Africa, where rapid and accurate pathogen identification can significantly influence disease control strategies [5].

Africa bears a disproportionate share of the global infectious disease burden, with recurrent outbreaks of viral, bacterial, and parasitic infections, including malaria, tuberculosis, HIV/AIDS, Ebola, and more recently, COVID-19 and mpox [6]. Limited laboratory infrastructure, inadequate funding, and workforce shortages challenge effective disease surveillance and response [7]. Strategies to combat infectious diseases on the continent increasingly emphasise strengthening laboratory networks, enhancing disease reporting systems, and leveraging technological advancements in genomics and bioinformatics [8]. Initiatives such as the Africa Pathogen Genomics Initiative (Africa PGI) under the Africa Centres for Disease Control and Prevention (CDC) aim to integrate genomics into national and regional disease surveillance frameworks [9]. However, capacity gaps in training and education remain a barrier to the widespread adoption of these tools [10].

The application of genomics and bioinformatics in pathogen surveillance provides several advantages. These tools facilitate early outbreak detection, antimicrobial resistance monitoring, and the development of targeted public health interventions [11]. In resource-limited settings, genomics enhances the accuracy of diagnostic tests and supports vaccine development by identifying circulating pathogen strains [8]. Furthermore, bioinformatics enables efficient data analysis and interpretation, transforming raw sequencing data into actionable epidemiological insights [12]. Despite these advantages, access to educational resources and training in genomics and bioinformatics remains limited in many African countries, hindering their full potential in public health [13].

Genomic epidemiology has become a cornerstone of modern pathogen surveillance, enabling real-time tracking of infectious agents [1]. Advances in next-generation sequencing (NGS) technologies have made it possible to rapidly sequence pathogen genomes at reduced costs, providing timely information for outbreak response [14]. Bioinformatics tools and computational pipelines are essential for processing and analysing genomic data, offering crucial insights into pathogen evolution and transmission dynamics [15]. The effective implementation of these technologies depends on a well-trained workforce, highlighting the need for comprehensive educational programmes and capacity-building initiatives across Africa [13].

Despite ongoing efforts to integrate genomics and bioinformatics into public health and clinical surveillance, access to educational materials and training opportunities in Africa is highly fragmented. Existing training programmes are often project-based, short-term, or geographically limited, leaving significant gaps in sustainable capacity development. Furthermore, many educational resources remain inaccessible due to financial and technical barriers [16, 17]. This fragmentation hinders the widespread application of these technologies in routine public health practice. To address these challenges, it is necessary to conduct a systematic review of available educational materials and training initiatives, identifying existing gaps and opportunities for improvement [18].

This scoping review aims to systematically map the landscape of training programmes related to the application of genomics and bioinformatics in pathogen surveillance in Africa. By identifying available capacity-building initiatives, highlighting deficiencies, and assessing accessibility, this review will provide a foundation for strengthening capacity-building efforts. The findings will inform policymakers, educators, and public health stakeholders, supporting the development of targeted training programmes that equip African scientists and public health professionals with the skills needed to harness genomics and bioinformatics for improved disease surveillance and outbreak response.

## Methods

### Review design and protocol

This scoping review was conducted following the *Preferred Reporting Items for Systematic Reviews and Meta-Analyses extension for Scoping Reviews* (PRISMA-ScR) guidelines [19]. A preliminary protocol was developed by the review team and agreed upon prior to screening and data extraction. The protocol was registered with the *Open Science Framework (OSF)* on the 3rd of December 2024 and updated on 7th April, 2025 (10.17605/OSF.IO/CT5H3*).* The objective was to map existing training initiatives in genomics and bioinformatics for pathogen surveillance in Africa, with a focus on identifying available resources, target audiences, gaps, and contextual relevance.

### Eligibility criteria

We included original studies (e.g., cross-sectional studies and case reports), reviews, perspectives, or opinion articles that focused on genomics or bioinformatics training for pathogen detection or environmental surveillance in Africa or targeting African populations. The review considered materials published in English between **November 2014 and December 2025**, capturing the most relevant and recent developments in this rapidly evolving field.

We excluded studies focused on plant or animal genomics, research on the human genome not linked to infectious disease surveillance, randomised controlled trials, and meta-analyses. The exclusion ensured that the scope remained focused on public health-relevant training related to pathogen genomics.

For the purpose of this review, included studies were categorised into two groups based on their primary focus. Training programme papers were defined as studies that described the design, implementation, delivery, or evaluation of a specific genomics and or bioinformatics training intervention, including workshops, courses, fellowships, or structured capacity-building programmes. Meanwhile, training needs or status papers were defined as studies that examined gaps, challenges, workforce capacity, or the broader landscape of genomics and bioinformatics training in Africa without reporting on a specific training intervention. These included opinion pieces, reviews, and cross-sectional assessments of capacity or training needs.

### Information sources and search strategy

A comprehensive and systematic literature search was conducted across the following electronic databases: PubMed, Embase, Scopus, Web of Science, OVID Global Health, and African Journals Online (AJOL). In addition, grey literature was explored through institutional contacts.

Search terms included combinations of keywords and controlled vocabulary, such as:

**“Genomics”**,** “Bioinformatics”**,** “Sequencing”**,** “Pathogen”**,** “Surveillance”**,** “Africa”**,** “Education”**,** “Training”**,** “Course”**,** “Handbook”**,** “Guideline”**.

Boolean operators (AND, OR) were used to enhance the search sensitivity and specificity. A librarian at the Oxford Bodleian Library was consulted to refine and validate the final search strategy.

#### PubMed search string

The final PubMed search string is as follows: (“genomics” OR “bioinformatics” OR “sequencing”) AND (“training” OR “education” OR “course” OR “workshop” OR “capacity building” OR “manual” OR “guideline” OR “curriculum” OR “program” OR “initiative”) AND (“pathogen” OR “infectious disease” OR “public health” OR “surveillance”) AND (“Algeria” OR “Angola” OR “Benin” OR “Botswana” OR “Burkina Faso” OR “Burundi” OR “Cabo Verde” OR “Cameroon” OR “Central African Republic” OR “Chad” OR “Comoros” OR “Congo” OR “Côte d’Ivoire” OR “Djibouti” OR “DR Congo” OR “Egypt” OR “Equatorial Guinea” OR “Eritrea” OR “Eswatini” OR “Ethiopia” OR “Gabon” OR “Gambia” OR “Ghana” OR “Guinea” OR “Guinea-Bissau” OR “Kenya” OR “Lesotho” OR “Liberia” OR “Libya” OR “Madagascar” OR “Malawi” OR “Mali” OR “Mauritania” OR “Mauritius” OR “Morocco” OR “Mozambique” OR “Namibia” OR “Niger” OR “Nigeria” OR “Rwanda” OR “São Tomé and Príncipe” OR “Senegal” OR “Seychelles” OR “Sierra Leone” OR “Somalia” OR “South Africa” OR “South Sudan” OR “Sudan” OR “Tanzania” OR “Togo” OR “Tunisia” OR “Uganda” OR “Zambia” OR “Zimbabwe”) AND (english[Language]) AND (“2014/11/01“[Date - Publication]: “2024/10/31“[Date - Publication]) Filters: English, from 2014 to 2025. The initial PubMed search string was registered, and it is available as an open-access search string [20]. Full details of the search strategies for each database are provided in Appendix 1.

The initial search was conducted on **6th of December 2024** and updated on **30th of March 2026**. Additional resources were sought through backward citation searching and expert consultation with members of the research team who are actively involved in genomics training programmes across Africa and key contacts in Africa CDC.

Grey literature was searched using the first 200 results from Google Scholar, and the WHO repository (https://www.who.int/) was also examined. Searches were conducted using combinations of the following keywords: (genomics OR bioinformatics OR sequencing) AND (training OR education OR workshop OR “capacity building” OR curriculum OR program) AND (pathogen OR “infectious disease” OR surveillance OR “public health”) AND (Africa OR African OR “each African country”).

### Selection of sources of evidence

The screening and selection process was conducted in two phases using Covidence systematic review software [21]. First, titles and abstracts were independently screened by five reviewers (PK, JM, PL, EN, JB) to assess eligibility based on the inclusion and exclusion criteria. Full texts of potentially eligible studies were then retrieved and reviewed for final inclusion by three reviewers (PK, JB, JM). Discrepancies were resolved through consensus. Duplicate entries were removed through the Covidence software. The entire review process involved extensive discussion, consensus building, and consultation with additional team members where necessary.

**Fig. 1:**
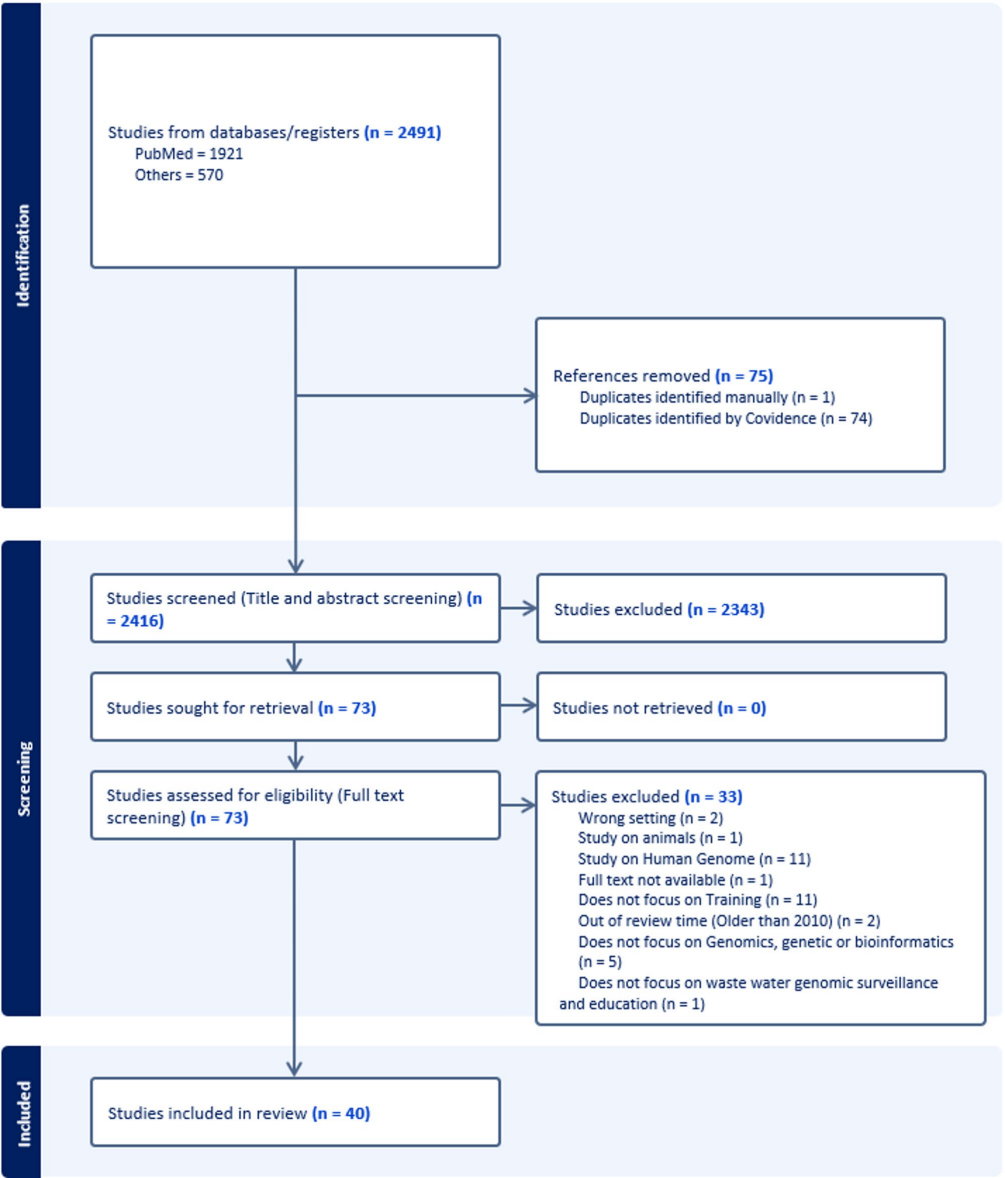
A PRISMA chart showing the article sources and screening process

The PRISMA flowchart was used to document the screening and selection process (Fig. 1).

### Data charting process

A data extraction template was developed collaboratively by the review team. It was piloted and refined prior to full extraction. The lead reviewer (PK) performed the initial charting, which was reviewed by JB to ensure completeness and consistency.

Extracted variables included:

- Study ID, author, title, year, and journal.
- Type of study or document (e.g., review, case report, training manual).
- Geographic setting or country focus.
- Study aim or purpose.
- Type and focus of training (genomics, bioinformatics, or both).
- Mode of delivery (online, in-person, hybrid).
- Duration and frequency of training.
- Target audience (e.g., researchers, students, healthcare workers).
- Reported outcomes of the training.
- Identified gaps or challenges.
- Recommendations for future capacity building.
- Organising institution(s) and funding source(s).

### Data analysis and synthesis

A narrative synthesis was conducted to summarise and categorise the findings. Descriptive statistics were used to illustrate frequencies, trends, and gaps in training provision across countries and thematic areas.

Findings were grouped according to themes such as:

- Geographic distribution of training programmes.
- Training content and alignment with public health needs.
- Accessibility (e.g., open-access vs. restricted).
- Equity and inclusivity in programme design and delivery.

This thematic analysis informed evidence-based recommendations to improve genomics and bioinformatics training in Africa.

## Results

The breakdown of the literature search is summarised in Fig. 1. Initial database searches conducted in December 2024 returned 2464 articles, this was updated in March 2026 generating 27 new articles. Out of the 2491 total records, 75 were duplicates, 2343 articles were excluded through both title and abstract screening and another 33 were excluded through full text review, resulting in 40 final included articles.

### Overview of included articles

We identified 40 publications, dated between 2014 and 2025, that explored training in genomics and bioinformatics across Africa (Table 1). The distribution of publications over the years was as follows: 2.5% (1 publication) in 2014 [22], 7.5% (3 publications) in 2015 [16, 17, 23], 12.5% (5 publications) in 2017 [24–28], 2.5% (1 publication) in 2018 [29], 7.5% (3 publications) in 2019 [30–32], 12.5% (5 publications) in 2020 [33–37], 12.5% (5 publications) in 2021 [38–42], 10% (4 publications) in 2022 [43–46], 12.5% (5 publications) in 2023 [47–51], 15% (6 publications) in 2024 [52–57], and 5% (2 publication) in 2025 [58, 59]. Case studies accounted for 45% (18 publications), opinion/perspective articles for 27.5% (11 publications), review articles for 15% (6 publications), cross-sectional studies for 10% (4 studies), and a methodological study with muti-site pilot validation (2.5%). Specific training programmes were evaluated or reported in 57.5% (23 publications), while 42.5% (17 publications) were included because they highlighted pathogen genomic/bioinformatics training needs or status in Africa (Table 1).

**Table 1:** Details of included articles

Study ID	Title	Year of publication	Journal/publisher	Study design	Reason for inclusion
Gilchrist 2015	Whole-Genome Sequencing in Outbreak Analysis.	2024	Journal ASM	Opinion/Perspective	Highlights training needs/status
DuarteASR 2020	Addressing Learning Needs on the Use of Metagenomics in Antimicrobial Resistance Surveillance.	2020	Frontiers in Public Health	Case Study	Reported a specific training
Chukwudi 2021	Consolidating and Upscaling Molecular Research Capacity in Nigeria: On Who’s Account?	2022	Frontiers in Research Metrics and Analytics	Opinion/Perspective	Highlights training needs/status
Davedow 2022	PulseNet International Survey on the Implementation of Whole Genome Sequencing in Low and Middle-Income Countries for Foodborne Disease Surveillance.	2022	FOODBORNE PATHOGENS AND DISEASE	Cross sectional study	Highlights training needs/status
Binder 2021	African National Public Health Institutes Responses to COVID-19: Innovations, Systems Changes, and Challenges.	2021	Health Security	Cross sectional study	Reported a specific training
Bentahir 2022	Providing On-Site Laboratory and Biosafety Just-In-Time Training Inside a Box-Based Laboratory during the West Africa Ebola Outbreak: Supporting Better Preparedness for Future Health Emergencies.	2022	International Journal of Environmental Research and Public Health	Case Study	Reported a specific training
Chimusa 2015	“Broadband” Bioinformatics Skills Transfer with the Knowledge Transfer Programme (KTP): Educational Model for Upliftment and Sustainable Development.	2015	PLOS Computational Biology	Case Study	Highlights training needs/status
Nembaware 2019	The African Genomic Medicine Training Initiative (AGMT): Showcasing a Community and Framework Driven Genomic Medicine Training for Nurses in Africa.	2019	Frontiers in Genetics	Case Study	Reported a specific training
deVries 2017	Regulation of genomic and biobanking research in Africa: a content analysis of ethics guidelines, policies and procedures from 22 African countries.	2017	BMC Medical Ethics	Cross sectional study	Highlights training needs/status
Musanabaganwa 2020	Building Skills and Resources for Genomics, Epigenetics, and Bioinformatics Research for Africa: Report of the Joint 11th Conference of the African Society of Human Genetics and 12th H3Africa Consortium, 2018.	2020	America Journal of Tropical Medicine	Case Study	Reported a specific training
Nyaga 2020	Report of the 1st African Enteric Viruses Genome Initiative (AEVGI) Data and Bioinformatics Workshop on whole-genome analysis of some African rotavirus strains held in Bloemfontein, South Africa.	2020	Vaccine	Case Study	Reported a specific training
MaljkovicBerry 2020	A Department of Defense Laboratory Consortium Approach to Next Generation Sequencing and Bioinformatics Training for Infectious Disease Surveillance in Kenya.	2020	Frontiers in Genetics	Case Study	Reported a specific training
HernÃ¡ndez-de-Diego 2017	The eBioKit, a stand-alone educational platform for bioinformatics.	2017	Computational biology	Review article	Reported a specific training
Carey 2023	Unlocking the Potential of Genomic Data to Inform Typhoid Fever Control Policy: Supportive Resources for Genomic Data Generation, Analysis, and Visualization.	2023	Open Forum Infectious Diseases	Review article	Highlights training needs/status
Shaffer 2019	Expanding Research Capacity in Sub-Saharan Africa Through Informatics, Bioinformatics, and Data Science Training Programmes in Mali.	2019	Frontiers in Genetics	Case Study	Reported a specific training
Marine 2019	Strengthening laboratory surveillance of viral pathogens: Experiences and lessons learned building next-generation sequencing capacity in Ghana.	2019	Elsevier, International journal of infectious diseases	Case Study	Reported a specific training
Gehre 2023	The East African Community mobile laboratory network prepares for monkeypox outbreaks.	2022	Journal of Public Health in Africa	Case Study	Reported a specific training
Tindana 2017	Developing the science and methods of community engagement for genomic research and biobanking in Africa.	2017	Global health, epidemiology and Genomics	Case Study	Reported a specific training
Giovanni 2023	African Centers of Excellence in Bioinformatics and Data Intensive Science: Building Capacity for Enhancing Data Intensive Infectious Diseases Research in Africa.	2023	Journal of Infectious Diseases and Microbiology	Case Study	Highlights training needs/status
Abrudan 2021	Train-the-Trainer as an Effective Approach to Building Global Networks of Experts in Genomic Surveillance of Antimicrobial Resistance (AMR).	2021	clinical infectious diseases, IDSA, Hivma, Oxford	Case Study	Reported a specific training
Ras 2021	Using a multiple-delivery-mode training approach to develop local capacity and infrastructure for advanced bioinformatics in Africa.	2021	Plos Computational Biology	Case Study	Reported a specific training
Ahmed 2020	Delivering blended bioinformatics training in resource-limited settings: a case study on the University of Khartoum H3ABioNet node.	2020	Briefings in Bioinformatics,	Opinion/Perspective	Reported a specific training
Gurwitz 2017	Designing a course model for distance-based online bioinformatics training in Africa: The H3ABioNet experience.	2017	Plos Computational Biology	Review article	Reported a specific training
Rivière 2021	Capacity building for whole genome sequencing of Mycobacterium tuberculosis and bioinformatics in high TB burden countries.	2021	Briefings in Bioinformatics	Case Study	Reported a specific training
Karikari 2015	Developing expertise in bioinformatics for biomedical research in Africa.	2015	Applied & Translational Genomics	Opinion/Perspective	Highlights training needs/status
Akintola 2024	Bioinformatics proficiency among African students.	2024	Frontier in bioinformatics	Opinion/Perspective	Reported a specific training
TastanBishopÃ– 2015	Bioinformatics education–perspectives and challenges out of Africa.	2014	Briefings in bioinformatics	Opinion/Perspective	Highlights training needs/status
Karikari 2015	Bioinformatics in Africa: The Rise of Ghana?	2015	Plos Comput	Opinion/Perspective	Highlights training needs/status
Kibet 2024	Designing and delivering bioinformatics project-based learning in East Africa.	2024	BMC Bioinformatics	Case Study	Reported a specific training
Sharaf 2024	Establishing African genomics and bioinformatics programmes through annual regional workshops.	2024	Nature genetics	Opinion/Perspective	Reported a specific training
Mulder 2017	Development of Bioinformatics Infrastructure for Genomics Research.	2017	gscience, Global heart	Review article	Highlights training needs/status
Pyden 2023	Teaching Infectious Disease Pathology and Taking it To Africa.	2023	Modern Pathology	Case Study	Reported a specific training
Onywera 2024	Boosting pathogen genomics and bioinformatics workforce in Africa.	2023	Lancet infectious disease	Opinion/Perspective	Reported a specific training
Osunmakinde 2018	Overview of Trends in the Application of Metagenomic Techniques in the Analysis of Human Enteric Viral Diversity in Africa’s Environmental Regimes.	2018	MDPI, Viruses	Review article	Highlights training needs/status
AbiaALK 2023	The African Wastewater Resistome: Identifying Knowledge Gaps to Inform Future Research Directions.	2023	MDPI, Antibiotics	Review article	Highlights training needs/status
Marklewitz 2025	Genomics costing tool: considerations for improving cost-efficiencies through cross scenario comparison	2025	Frontiers in Public Health	Methodological study with multi-site pilot validation	Highlights training needs/status
Mboowa 2024	The rise of pathogen genomics in Africa	2024	*F1000 Research*	Opinion/Perspective	Highlights training needs/status
Inzaule 2021	Genomic-informed pathogen surveillance in Africa: opportunities and challenges	2021	The Lancet Infectious Diseases	Opinion/Perspective	Highlights training needs/status
Nguinkal 2024	Assessment of the pathogen genomics landscape highlights disparities and challenges for effective AMR surveillance and outbreak response in the East African Community	2024	BMC Public Health	Cross sectional study	Highlights training needs/status
Agboli 2025	Building Pathogen Genomic Sequencing Capacity in Africa: Centre for Epidemic Response and Innovation Fellowship	2025	Tropical Medicine and Infectious Disease (MDPI)	Case Study	Reported a specific training

### Characteristics of the identified training programmes

A total of 23 articles reported or evaluated specific training programmes.

**Fig. 2:**
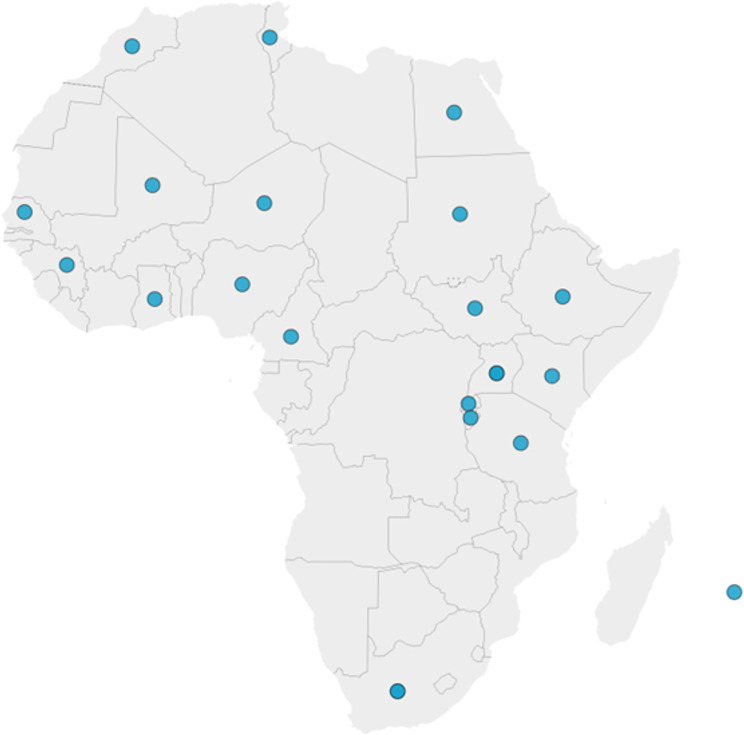
Host countries of included genomic/bioinformatics training

**Fig. 3:**
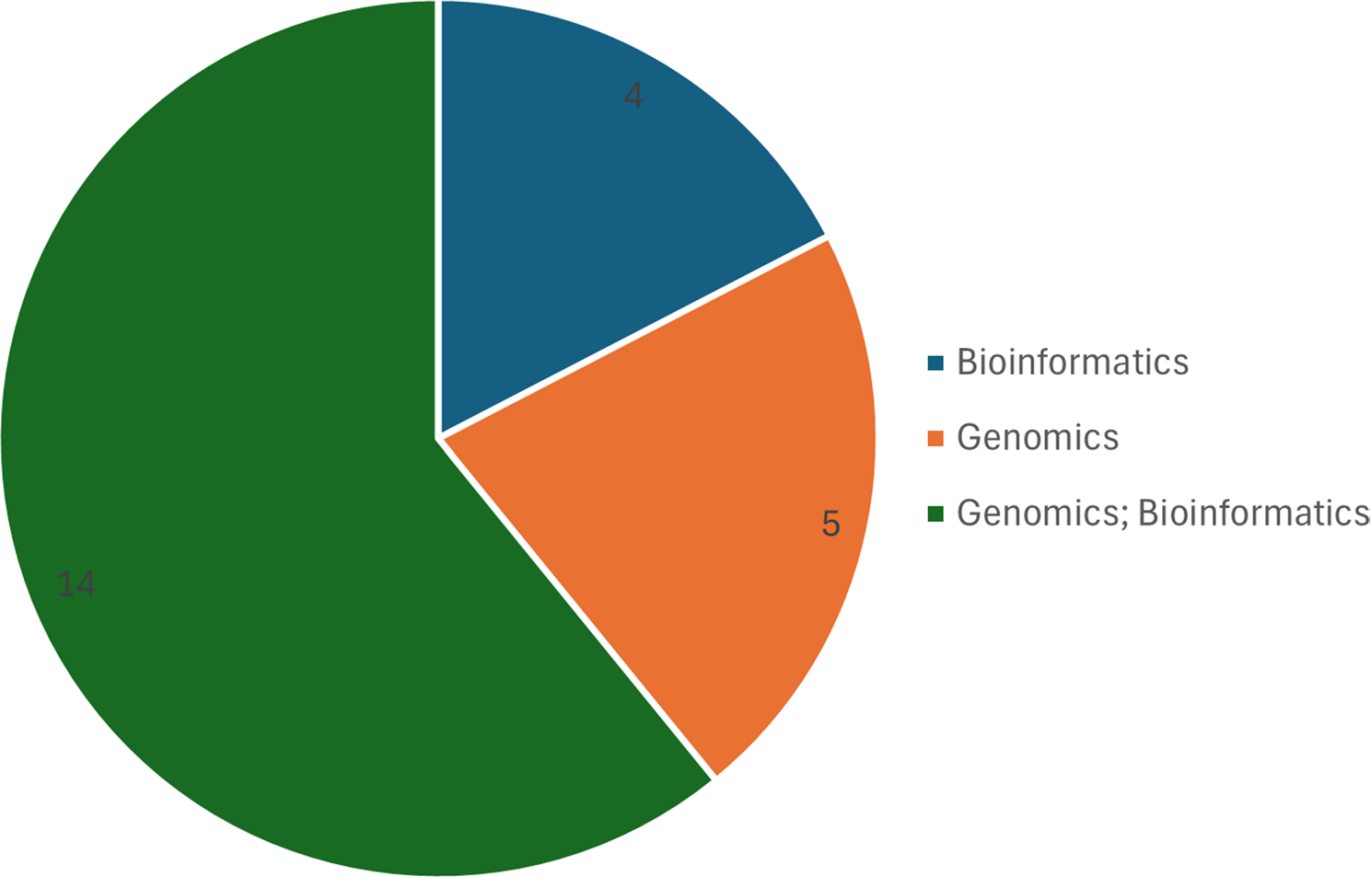
The Focus of the training programmes

Between 2014 and 2024, the highest number of training programmes occurred in 2019 and 2023 (4/23 each, 17.3%), followed by 2017 (3/23, 13%). Only one programme each was recorded for 2016 and 2022 (4.3%). Additionally, one training programme (4.3%) reported ongoing training activities since 2007. In terms of location, 21 of the 23 training programmes (91.3%) were held or hosted across different countries in Africa, while one programme (4.3%) was hosted in the United Kingdom and another (4.3%) in the United States (Fig. 2). Five training programmes (21.7%) addressed genomics alone, 17.4% (4 programmes) focused solely on bioinformatics, and 60.9% (14 programmes) covered both genomics and bioinformatics (Fig. 3). Training duration varied considerably. Specifically, 13% (3 programmes) lasted between two and five days, 17.4% (4 programmes) lasted one to two weeks, 4.3% (1 programme) lasted three weeks, 4.3% (1 programme) lasted eight weeks, 8.7% (2 programmes) lasted two to four months, 4.3% (1 program) lasted 21 weeks, and 4.3% (1 programme) lasted two years. Notably, 43.5% (10 programmes) did not specify the duration.

The nature of training delivery also varied: 60.9% (14 programmes) were delivered in person, 13% (3 programmes) online, 8.7% (2 programmes) in hybrid formats, 8.7% (2 programmes) as blended learning, and 8.7% (2 programmes) were unspecified. In terms of frequency, 43.5% (10 programmes) were offered as one-off events, 34.8% (8 programmes) were delivered regularly (either yearly or monthly), and 8.7% (2 programmes) were ongoing.

Finally, regarding the online availability of resources, 26.1% (6 programmes) made materials available online, 4.3% (1 programme) offered on-demand access, while 69.6% (16 programmes) did not provide access to training resources online. Full details of the 23 articles that reported or evaluated specific training programmes are provided in Appendix 2.

#### Training outcomes

Among the 23 articles that reported on training programmes, 13 (56.5%) reported specific training outcomes. Most reported training outcomes using self-reported measures, with relatively few employing objective indicators such as completion rates, career progression, or research outputs. The most common outcomes included enhanced technical skills (5, 21.7%) and career development (4, 17.4%). Other outcomes reported include increased participation through virtual formats (13%), strengthened professional networks (13%), successful project implementation (8.7%), and institutional capacity building (8.7%) (Table 2).

**Table 2:** Reported outcomes of training programmes (*n* = 23)

Outcome Category	Number of programmes (%)	Example Statement
Skill Improvement	5 (21.7%)	*“There were substantial gains in self-reported knowledge across all skill domains.” (MaljkovicBerry*,* 2020)*
Career Development	4 (17.4%)	*“The training had a significant impact on the career development of the two Guinean biologists.” (Bentahir*,* 2022)*
Increased Participation and Reach	3 (13%)	*“The transition to a virtual format during the pandemic allowed the program to accommodate a larger number of participants.” (Kibet*,* 2024)*
Network and Community Building	3 (13%)	*“This course provided a unique opportunity for connecting young scientists across Africa.” (Gurwitz*,* 2017)*
Project Implementation	2 (8.7%)	*“Many participants engaged in collaborative mini-projects*,* which were noted as a highlight of the training.” (Kibet*,* 2024)*
Institutional Capacity Building	2 (8.7%)	*“The primary outcomes of the ITMI and ITGH training programmes included numbers of college degrees earned and publication frequencies and rates.” (Shaffer*,* 2019)*
Funding Success	1 (4.3%)	*“Within 1 year of training*,* nine trainees submitted a fundable proposal.” (Riviere*,* 2021)*
Positive Feedback	1 (4.3%)	*“A survey conducted during the training indicated that participants were satisfied with the training modules*,* quality of presentations*,* and overall experience.” (Akintola*,* 2024)*

#### Training organisers and funders

The 23 training programmes were organised by a range of entities. Specifically, 21.7% (5 programmes) were organised by the Pan African Bioinformatics Network (H3ABioNet), while 8.7% (2 programmes) were organised by the African Society of Human Genetics (AfSHG) in collaboration with other partners.

Regarding funding sources, 26.1% (6 programmes) were funded by the United States National Institutes of Health, 13% (3 programmes) by the Wellcome Trust, and 8.7% (2 programmes) by the United States CDC. In addition, 4.3% (1 programme each) received funding from the Bill and Melinda Gates Foundation, the European Commission, the European Space Agency’s Integrated Applications Promotion Advanced Research in Telecommunications Systems (ARTES) programme, the German Federal Ministry for Economic Cooperation and Development (BMZ) through the German Development Bank (KfW), a consortium involving the Government of Rwanda, Institut Pasteur, Promega Corporation, Africa Biosystems Limited, and The Galton Institute, the United Kingdom National Institute for Health Research, the Renal Pathology Society, the United States Government and Department of Defense, and VLIR-UOS. Another training initiative was funded by a consortium of Abbott Pandemic Defence Coalition, WHO AFRO, Africa CDC, and Rockefeller Foundation. Additionally, 26.1% (6 programmes) did not specify their sources of funding.

#### Challenges to training

Key challenges identified from the 23 training programmes with example quotes are as below.

- Infrastructure and resource limitations:
  - DuarteASR (2020): “Uneven access of learners to infrastructures (internet bandwidth, computer processor, operating system and memory) will impact on the learning outcome and the likelihood of course completion.”
  - Ras (2021): “One of the main challenges with this approach is the computational requirements for the hosting site. Some classrooms could not participate as they could not meet classroom specifications.”
- Skill gaps and participant preparedness:
  - Riviere (2021): “We experienced that trainees were often underprepared for basic bioinformatics instruction.”
  - MaljkovicBerry (2020): “There were substantial gains in self-reported knowledge across all skill domains, with the notable exception of Linux OS and command line skills, suggesting that this is a particular area of residual training need.”
  - Kibet (2024): “In hybrid settings, having participants with varying levels of expertise sometimes forced trainers to recap introductory content, which could disadvantage more advanced students.”
- Content delivery and curriculum design:
  - Kibet (2024): “Participants noted that the training had limited practical components due to the time constraints of the modules, leading to a greater focus on theoretical aspects.”
  - Ras (2021): “Several participants indicated that the introductory modules were too short and suggested these modules be increased in length.”
  - Gurwitz (2017): “The availability of pre-recorded lectures…provided the initial step for the possible adoption of a flipped classroom model…[but] local staff would need to be adequately trained on the approach.”
  - Agboli (2025): “Insufficient time… particularly for the computationally intensive bioinformatics sessions.”
- Sustainability and long-term engagement:
  - Akintola (2024): “The retention of bioinformatics talent in Africa is a concern, as there is a potential for brain drain.”
  - Riviere (2021): “We struggled to maintain long-term commitment from the trainees after the trainings.”
  - Shaffer (2019): “Long-term sustainability of training and capacity development in Africa will likely require additional support within the host countries in the area of research and development.”

#### Training opportunities and enablers grouped into themes with quotes

The training opportunities and enablers identified from the 23 training programmes are as follows:

- Innovative delivery models:
  - Gurwitz (2017): “By ensuring redundancy (e.g., live question and answer sessions via video conferencing sessions as well as question and discussion forums), we could ensure that internet instability was not a barrier to education.”
  - Kibet (2024): “The COVID-19 pandemic necessitated a shift to virtual training, which provided an opportunity to reach a wider audience.”
  - Ahmed (2020): “Delivering blended bioinformatics training facilitated by local TAs…gave participants clear expectations, and they were able to both evaluate their progress and meet the course requirements.”
- Collaborative networks and partnerships:
  - Kibet (2024): “The training fostered strong community ties among participants, leading to continued collaboration after the course ended.”
  - Akintola (2024): “Collaborative efforts between African universities and international institutions would ensure progress in training students and researchers in bioinformatics.”
  - Onywera (2024): “The training workshops have…increased their continental and global recognition, which improves their potential for collaboration with public health, academic, and research institutions.”
  - Agboli (2025): “[The training] provided a springboard… to launch collaborative projects.”
- Hands-on and project-based learning:
  - Kibet (2024): “The program emphasized project-based and hands-on learning, which has been noted to effectively prepare students for real-world research scenarios.”
  - Nyaga (2020): “The workshop fostered interactions and networking between professionals, scientific experts, technicians and students.”
  - MaljkovicBerry (2020): “The participants also reported that the information on study design, sample preparation, sequencing quality control, data quality assessment, reporting, and basic and advanced bioinformatics analysis were the most useful.”
- Capacity building and local empowerment:
  - Gurwitz (2017): “As several of the volunteer staff were PhD candidates or postdoctoral students…this course provided a platform for education-capacity development.”
  - Shaffer (2019): “Training programmes are perhaps best facilitated through Africa’s university systems as they are perhaps best positioned to maintain core resources during lapses in short-term funding.”
  - Bentahir (2022): “The training had a significant impact on the career development of the two Guinean biologists.”
- Online resources and open access:
  - Ras (2021): “Content remains available indefinitely after course end and are under a creative commons license allowing anyone to reuse it with the proper credit.”
  - Akintola (2024): “Getting the instructors to deliver virtual lectures is no longer a problem as there are hundreds of free bioinformatics resources available online.”
  - Hernandez-de-Diego (2017): “The eBioKit…has self-contained courses and tutorials, teaching both basic and advanced bioinformatics using software and databases installed locally on the platform.”

### Thematic analysis of training deficiencies, opportunities, and recommendations with quotes

Thematic analysis of the 17 articles highlighting training needs or discussing the state of genomic or bioinformatics capacity for pathogen surveillance in Africa identified key themes for deficiencies, opportunities, and recommendations, with supporting quotes.

#### Deficiencies in training

Limited infrastructure and resources:

- Karikari (2015, Ghana): “Effective bioinformatics applications require essential resources such as powerful computer systems, high-speed internet connectivity, and continuous electrical power supply. In Ghana, these resources are not usually available.”
- Mulder (2017): “Challenges identified include slow and unstable Internet connectivity; unreliable power supply; continent-wide obsolete computer infrastructure.”
- Osunmakinde (2018): “The relatively high cost of modern molecular technologies, as well as computational human expertise for the analysis of the data generated, have greatly contributed to the slow growth of the viral microbial ecological research community in Africa.”

Shortage of trained personnel and expertise:

- Chukwudi (2021): “In the earlier days of molecular research in Nigeria, one of the major challenges was that of having adequately trained manpower.”
- TastanBishop (2015): “The main reason is most likely the lack of academic faculty able to supervise purely bioinformatics research projects in some of the African institutions.”
- Davedow (2022): “The primary challenges and barriers to WGS implementation are related to access to qualified personnel and data analysis and interpretation.”
- Mboowa (2024): “Majority of the workforce has limited experience in these fields largely due to the limited number of training institutions…” “There is an urgent need… to recruit and retain competent personnel dedicated to performing bioinformatics analyses…”.
- Nguinkal (2024): “[There is] need for more trained staff in NGS data analysis, including pipelines and workflow deployment.”
- Inzaule (2021): “There is a need to develop local expertise for equipment installation and maintenance.”

Inadequate training programmes and curricula:

- Karikari (2015, Ghana): “Whilst a few institutions have some scientists with appreciable knowledge in bioinformatics, most Ghanaian institutions lack this human resource capacity. This lack of expertise may adversely affect the quality of training.”
- TastanBishop (2015): “There is no unified Masters bioinformatics curriculum within Africa.”
- AbiaALK (2023): “Metagenomics, though more comprehensive, is rarely used due to high costs and limited technical expertise, particularly in bioinformatics.”

Brain drain and retention challenges:

- Chukwudi (2021): “The challenges of non-functional laboratories and poor or unconducive working environment are now driving the emigration of such skilled personnel to the technologically advanced countries.”
- Chimusa (2015): “A substantial number of African scientists, including bioinformaticians, go abroad because of competitive salaries, better job opportunities, and well-resourced institutions.”
- Karikari (2015, Ghana): “The short supply of expertise remains a major challenge.”
- Marklewitz (2025): “…offering competitive salaries and implementing contractual regulations are essential for sustaining investments in human resources”.

#### Opportunities for training

Short-term training and workshops:

- Karikari (2015, Biomedical): “The following organisations regularly organise bioinformatics and the African scientists: ASBCR, ISCR, H3ABioNet.”
- TastanBishop (2015): “In addition to degree programmes, many short training workshops are offered throughout Africa, up until recently, mostly organized and sponsored by foreign institutions.”
- Chukwudi (2021): “The National Institutes of Health (NIH), in partnership with Wellcome Trust, initiated a 10-year research funding (2011–2021) for the Human Heredity and Health in Africa (H3Africa) project to support African institutions.”

Formal academic programmes:

- Karikari (2015, Biomedical): “Bioinformatics degree programmes have been introduced in different countries, including Egypt, Mauritius, Mali, Kenya, Nigeria, South Africa, and Tunisia”.
- TastanBishop (2015): “Most of the bioinformatics programmes offered at Masters level have a component of course work and lectures to accommodate students with different educational backgrounds.”
- Chimusa (2015): “The KTP aims to develop a network with a centralized hub through which knowledge seekers from African institutes or companies and knowledge providers or experts from around the world are put into contact with one another.”

Collaborative initiatives and networks:

- Karikari (2015, Ghana): “Developing collaborations between Ghanaian scientists could be a useful mechanism to build bioinformatics expertise.”
- TastanBishop (2015): “H3ABioNet is also training students through involvement in joint research projects relevant to the goals of the network.”
- Chukwudi (2021): “The NIH, in partnership with Bill and Melinda Gates Foundation and the African Academy of Science, is taking the lead with the African Postdoctoral Training Initiative (APTI).”
- Mboowa (2024): “Existing initiatives like AI4D Africa, H3AbioNet, EANBiT, African BioGenome Project and several university training programmes provide a strong foundation…”.
- Inzaule (2021): “A network approach across Africa would help use the few resources available.”

Open-source and E-Learning resources:

- Karikari (2015, Ghana): “One advantage of bioinformatics is the availability of several free, open-source resources (FOSR). FOSR allow for a virtual learning model of education to be applied to bioinformatics.”
- TastanBishop (2015): “E-learning platforms provide materials and computer-assisted courses that involve hands-on practical exercises.”
- Davedow (2022): “With WGS comes universal, unambiguous, and comparable data generated by all and the opportunity to enable global foodborne disease surveillance.”

#### Recommendations

Enhance infrastructure and funding:

- Chukwudi (2021): “It is high time the government steps up with a definite research policy and agenda setting to give research direction to researchers. This cannot be achieved without the government prioritizing research and improving research funding.”
- AbiaALK (2023): “Funding must be made available to researchers as sequencing technologies are not yet widespread in the country, and the cost of using these facilities is still considerably high.”
- Carey (2023): “Governments, donors, and other stakeholders continue to support the establishment and sustenance of molecular surveillance capacity by providing funding for procurement of equipment and reagents.”
- Nguinkal (2024): “Establishing local bioinformatics centers… can be hubs for bioinformatics training.”

Expand training programmes and curricula:

- Karikari (2015, Ghana): “Degree programmes and other forms of advanced training (such as research fellowships) will also be essential to provide learners with in-depth skills in bioinformatics.”
- TastanBishop (2015): “A system of continuing mentorship and support would ensure that students are able to have regular contact with trainers.”
- AbiaALK (2023): “There is a need to build capacity in sequencing technologies and bioinformatics, given the recent drift of the science to big data analysis.”

Foster collaborations and networks:

- Karikari (2015, Ghana): “Developing collaborative research efforts between scientists in Ghana and their colleagues elsewhere in Africa may also be a sustainable means to boost bioinformatics activities.”
- Chimusa (2015): “We propose a formalized matchmaking system, which is aimed at reversing this trend, by introducing the Knowledge Transfer program (KTP).”
- Chukwudi (2021): “They should continue to collaborate with both local and foreign colleagues to achieve their research aims.”
- Mboowa (2024): “Establish collaborative training programmes through partnerships between African institutions and international institutions…”.

Address Brain Drain and Retention:

- Chukwudi (2021): “The research institutions need to step up efforts to provide the necessary support to the researchers by providing a conducive working environment for them.”
- Karikari (2015, Biomedical): “African governments and policy makers need to be educated about the value of their research, and the need to invest further in it.”
- Chimusa (2015): “The KTP aims to develop and transfer hands-on experience in analysing data from various current proteomic, genomic, and other platforms, using cutting-edge techniques.”
- Inzaule (2021): “Innovative incentives… might be needed to retain the diverse genomics workforce.”

## Discussion

This scoping review mapped training programmes in genomics and bioinformatics across Africa, with a specific lens on their application to pathogen surveillance. The 40 identified articles, spanning 2014 to 2025, reveal a growing yet uneven landscape of capacity-building efforts, offering insights into available opportunities, delivery mechanisms, and persistent gaps. These findings align with our objective to systematically review resources and identify deficiencies, providing a foundation for strengthening surveillance capabilities in a region where infectious diseases pose significant public health challenges. Our analysis reveals several critical trends and gaps, while also identifying opportunities for strengthening capacity.

A predominant feature of genomics and bioinformatics training in Africa is its short-term, ad hoc nature. Most initiatives identified in our review were one-off events, often held at the fringes of conferences and lasting from a few days to weeks. While such interventions provide foundational exposure, they are unlikely to foster the depth of expertise or sustainability required to build a critical mass of skilled professionals. This contrasts sharply with approaches in other regions. In Europe and North America, similar programmes span several months to years and are frequently embedded in academic institutions, such as the bioinformatics training portfolio of The European Bioinformatics Institute of the European Molecular Biology Laboratory (EMBL-EBI), which integrates short courses, summer schools, and online courses to reinforce learning over time [60]. Worthy of note, the Collaborative African Genomics Network (CAfGEN), although Africa-based, has adopted a two-year continuous programme incorporating experiential learning, demonstrating that long-term models are feasible even within the continent. According to Shaffer et al. (2019) while short-term training has some benefits, “Long-term, incremental processes are necessary” for meaningful engagement in bioinformatics and data science research. This approach could shift training from episodic to structured and continuous models [31]. In addition to extending the duration of training, structured curricula should also integrate components on global (meta)data standards and FAIR principles. Embedding modules on reproducible workflows (e.g. Galaxy, Snakemake, Nextflow) and metadata reporting would ensure that trainees not only gain technical skills but also learn how to generate outputs that are interoperable with international data infrastructures. Such alignment would strengthen the long-term sustainability and reusability of African pathogen surveillance data and avoid the fragmentation that has limited adoption in other domains.

Furthermore, only about a quarter of the training programmes in our review made resources available online. This presents a significant barrier to sustained learning, especially in a context where in-person opportunities are scarce and digital self-learning could be a powerful alternative. Globally, training repositories like Coursera, edX, and the OpenWHO platform provide freely accessible, high-quality genomics and bioinformatics courses [61]. Within Africa, H3ABioNet stands out for its investment in online training resources, including massive online open courses (MOOCs) and e-learning modules [13]. The underutilisation of open-access platforms by other programmes is a missed opportunity that could be addressed through the adoption of Creative Commons licensing, as demonstrated by Ras (2021), whose content remained freely available beyond the training period [40]. To maximise impact, however, online materials must also be designed for accessibility in low-resource environments, for instance through lightweight platforms, offline-compatible modules, or regionally hosted resources. Ensuring that training resources remain usable across diverse African contexts is essential, as even high-quality content has limited value if it cannot be reliably accessed.

An overwhelming proportion of the programmes were funded by external agencies such as the United States NIH, the Wellcome Trust, and the United States CDC. While this funding has been pivotal in jump-starting many initiatives, the limited contribution of African governments and local institutions raises questions about long-term sustainability. In contrast, countries such as India have successfully leveraged national funding mechanisms through bodies like the Department of Biotechnology to support graduate and postgraduate bioinformatics programmes [18]. Encouragingly, some African governments (e.g. Rwanda) have begun to co-invest in training efforts, but broader commitment is needed across the continent [53]. Such investment should also be tied to national strategies that emphasise integration with international data infrastructures (e.g. ENA), ensuring that publicly funded training prepares scientists to contribute interoperable, globally relevant data.

When outcomes were reported, they included improved technical skills, career progression, enhanced participation (especially in virtual formats), and the development of collaborative networks. These results align with those from similar global initiatives, such as the Wellcome Genome Campus Advanced Courses, which have documented career impact through alumni tracking [62]. Notably, the Guinean biologists trained through a Wellcome-funded programme achieved significant career growth, and a virtual training shift during COVID-19 increased accessibility and reach [45, 54]. However, the lack of consistent metrics for assessing long-term impact limits the ability to draw broad conclusions. Systematic monitoring and evaluation tools, as employed by programmes in the Global North, are crucial for benchmarking progress and guiding future investments. Additionally, future evaluations should not only assess individual career progression but also compare the broader contributions of trained versus non-trained scientists, for instance in terms of publications, use of open pipelines, or submissions to regional and international databases.

The evolving landscape of genomics and bioinformatics training in Africa is increasingly shaped by a diverse ecosystem of stakeholders, including international organisations, regional public health bodies, academic institutions, and private sector partners. Initiatives involving organisations such as the WHO, Africa CDC, and regional laboratory networks have played a pivotal role in supporting training delivery, funding, and infrastructure development. For example, recent training programmes have been implemented through multi-partner collaborations involving Africa CDC, WHO regional offices, and research consortia, reflecting a growing emphasis on coordinated capacity building efforts [59]. At the same time, continental initiatives such as H3ABioNet, the African BioGenome Project, and regional training networks provide important platforms for knowledge exchange and workforce development [56]. While these partnerships have significantly expanded access to training opportunities, the landscape remains fragmented, with variability in programme design, sustainability, and alignment with national priorities. Strengthening coordination across stakeholders, alongside greater leadership from African governments and institutions, will be essential to ensure that training investments are harmonised, contextually relevant, and capable of supporting long-term surveillance capacity.

This review highlights several persistent challenges that hinder the effectiveness of bioinformatics and genomics training across Africa. One of the most pressing issues is inadequate infrastructure. Many institutions report poor internet connectivity, unstable electricity supply, and outdated hardware, all of which constrain both online and in-person training efforts [37, 53]. Compounding this is the heterogeneity in participant backgrounds. Trainees often enter programmes with varying levels of preparedness, which necessitates repeated coverage of introductory material and limits the progression of more advanced learners [34, 54]. Furthermore, time constraints commonly lead to curricula that are heavily theoretical, with limited opportunities for practical, hands-on training. This imbalance hampers the development of applied skills that are essential in real-world research contexts. Additionally, there is a marked shortage of qualified local faculty. The limited availability of trainers and mentors with relevant expertise compromises the continuity and scalability of training programmes, particularly in institutions without established bioinformatics departments [22]. Nevertheless, promising developments are emerging within Africa. H3ABioNet’s train-the-trainer model, for example, illustrates how local capacity can be built through the strategic training of regional instructors who then cascade knowledge within their home institutions. Looking ahead, training programmes should also anticipate the growing need to handle increasingly heterogeneous data types, ensuring that participants are equipped to integrate genomic information with other epidemiological and laboratory data sources used in pathogen surveillance.

An important systems-level challenge emerging from recent evidence is the mismatch between expanding genomic infrastructure and the availability of adequately trained personnel to utilise it effectively. While investments in sequencing technologies and laboratory capacity have increased across parts of Africa, workforce development has not kept pace, resulting in underutilisation of available resources. For instance, studies have highlighted that a significant proportion of genomic data generated within African settings continues to be processed and analysed externally, reflecting persistent gaps in local bioinformatics expertise [57]. Similarly, broader analyses emphasise that insufficient numbers of trained personnel and challenges in retaining skilled staff limit the efficiency and sustainability of genomic surveillance systems [58]. This imbalance suggests that technological advancement alone is insufficient; parallel and sustained investment in human capacity is essential to ensure that infrastructure translates into meaningful public health impact.

Despite these obstacles, several enablers and opportunities have emerged that can support the effective development of bioinformatics and genomics training across the continent. Innovative delivery models, such as hybrid and virtual training formats introduced during the COVID-19 pandemic, have demonstrated substantial potential for expanding reach and participation [33]. Furthermore, regional collaborative networks such as H3ABioNet and the African Society of Human Genetics (AfSHG) have played a crucial role in building communities of practice, fostering peer support, and facilitating cross-border knowledge exchange [28]. The incorporation of hands-on learning and mini-projects into training programmes has proven particularly effective in reinforcing technical skills and enabling participants to apply their knowledge to real-world problems [54]. In addition, open-access resources such as the eBioKit platform offer offline access to training content, which is especially valuable in settings where internet bandwidth is limited [26]. Internationally, initiatives such as India’s Bioinformatics National Certification programme and Brazil’s Fiocruz Genomics School provide compelling models of how government coordination and public–private partnerships can successfully scale bioinformatics education to align with national health research priorities [63, 64]. Building on these opportunities, African training efforts could also embed (meta)data standards, FAIR practices, and open science principles from the outset, ensuring that capacity development grows on a foundation of sustainability and interoperability.

A critical gap identified across the literature is the limited evidence linking training initiatives to tangible improvements in pathogen surveillance systems. While many programmes report gains in technical skills and increased confidence among participants, there remains insufficient documentation of whether these competencies translate into sustained application within national public health systems. Recent evidence highlights that, despite investments in sequencing infrastructure, a substantial proportion of genomic data analysis in some African regions continues to be conducted by external partners, reflecting persistent gaps in local analytical capacity [57]. This suggests that training efforts, in their current form, may not yet be fully embedded within operational surveillance frameworks. Similarly, calls for developing a critical mass of locally trained experts underscore the importance of not only expanding training opportunities but ensuring that these are aligned with workforce retention and institutional integration [42]. Bridging this gap requires a shift from short-term capacity building towards longitudinal training models that are closely linked to health system needs, supported by mentorship, and reinforced through opportunities for practical application. Future training programmes should therefore incorporate mechanisms for tracking downstream impact, such as contributions to national surveillance outputs, implementation of genomic workflows, and integration into routine outbreak response activities.

### Limitations

Several limitations were anticipated and carefully mitigated in this review. First, our protocol excluded non-English studies and literature published before 2014. While this may have limited the inclusion of francophone African resources or earlier foundational programmes, we sought to minimise the impact by conducting an extensive search across diverse databases and grey literature sources, ensuring broad geographical and thematic coverage. In addition, engagement with domain experts and review of reference lists helped capture significant initiatives that might otherwise have been missed.

### Recommendations

To strengthen the quality, sustainability, and long-term impact of bioinformatics and genomics training in Africa, several strategic actions are recommended. Firstly, training must be institutionalised within academic curricula to enable credentialing and career progression for trainees. This institutionalisation should not only cover technical genomic and bioinformatics skills but also explicitly include training in FAIR data principles, global (meta)data reporting standards, and reproducible workflow tools. Embedding these practices early will ensure that African trainees are prepared to generate outputs that can be seamlessly integrated with international data infrastructures. Secondly, programme developers should prioritise the publication of training materials under open licences, thereby facilitating continued access and reuse across the continent. To ensure equity, training resources should also be designed for low-resource environments, for instance through offline-compatible modules or lightweight platforms, so that online learning opportunities are usable across diverse African contexts. Thirdly, national governments must play a more proactive role by increasing investment in genomics education through dedicated policy frameworks and budget allocations. Fourthly, robust monitoring and evaluation mechanisms should be embedded in all training programmes to assess their effectiveness and inform continuous improvement. Evaluations should not only track career progression but also capture systemic contributions, such as the adoption of open pipelines, FAIR-compliant datasets, or submissions to regional and international databases. Finally, local trainer development should be prioritised to ensure that African institutions have the human resources necessary to sustain high-quality, contextually relevant education independently of external actors.

## Conclusion

While genomics and bioinformatics training for pathogen surveillance in Africa has expanded considerably over the past decade, it remains characterised by short-term, externally funded initiatives that are not yet fully integrated into national health systems. This review highlights that, beyond increasing the availability of training programmes, there is a critical need to align capacity building efforts with workforce retention, infrastructure development, and the operational demands of public health surveillance. Persistent gaps in local analytical capacity, including continued reliance on external partners for genomic data analysis, underscore the importance of transitioning from episodic training models to sustained, system-oriented approaches. Strengthening the impact of training will require embedding programmes within academic and public health institutions, fostering long-term mentorship, and implementing robust evaluation frameworks that capture downstream contributions to surveillance and outbreak response. In addition, improved coordination among key stakeholders, including governments, regional bodies, and international partners, will be essential to ensure that investments in training translate into resilient and self-sustaining surveillance systems. With strategic alignment and sustained commitment, Africa is well positioned to develop a skilled workforce capable of harnessing genomics and bioinformatics to enhance infectious disease preparedness and response.

## Supplementary Information

Below is the link to the electronic supplementary material.

Supplementary Material 1: The Search Strategy

Supplementary Material 2: Details of the 23 training programmes identified

Supplementary Material 3: The complete dataset

## Data Availability

All data generated or analysed during this study are included in this published article as supplementary material (Appendix 3).

## Abbreviations

AfSHG: African Society of Human Genetics
AJOL: African Journals Online
CDC: Centres for Disease Control and Prevention
EMBL-EBI: The European Bioinformatics Institute of the European Molecular Biology Laboratory
KTP: Knowledge Transfer program
MOOC: massive online open courses
NGS: next-generation sequencing
NIH: National Institutes of Health
PGI: Pathogen Genomics Initiative
PRISMA-ScR: Preferred Reporting Items for Systematic Reviews and Meta-Analyses extension for Scoping Reviews
WGS: whole genome sequencing
WHO: World Health Organisation
